# The Origins & Reservoirs of Exocomets

**DOI:** 10.1007/s11214-025-01219-w

**Published:** 2025-09-29

**Authors:** Michele Bannister, Susanne Pfalzner, Tim Pearce, Alexander J. Mustill, Hubert Klahr, Hideko Nomura, Nagayoshi Ohashi, Rosita Kokotanekova, Sebastian Marino, Dennis Bodewits, Raphael Marschall, Darryl Z. Seligman, Geraint H. Jones, Dimitri Veras

**Affiliations:** 1 School of Physical and Chemical Sciences – Te Kura Matū, University of Canterbury, Private Bag 4800, Christchurch, 8140 New Zealand; 2 Jülich Supercomputing Centre, Research Centre Jülich, Wilhelm-Johnen-Strasse, Jülich, 52428 Germany; 3 Department of Physics, University of Warwick, Gibbet Hill Road, Coventry, CV4 7AL United Kingdom; 4 Department of Physics, Lund University, Box 118, Lund, 22100 Sweden; 5 Max Planck Institut für Astronomie, Königstuhl 17, Heidelberg, 69117 Germany; 6 Division of Science, National Astronomical Observatory of Japan, 2-21-1, Osawa, Mitaka, Tokyo 181-8551 Japan; 7 Academia Sinica Institute of Astronomy & Astrophysics, 11F of Astronomy-Mathematics Building, AS/NTU, No.1, Sec. 4, Roosevelt Rd, Taipei, 106319 Taiwan, R.O.C.; 8 Institute of Astronomy and NAO, Bulgarian Academy of Sciences, 72 Tsarigradsko Chaussee Blvd., Sofia, 1784 Bulgaria; 9 International Space Science Institute, Hallerstrasse 6, Bern, 3012 Switzerland; 10 Department of Physics and Astronomy, University of Exeter, Stocker Road, Exeter, EX4 4QL UK; 11 Department of Physics, Auburn University, Edmund C. Leach Science Center, Auburn, 36849 AL USA; 12 Laboratoire J.-L. Lagrange, Observatoire de la Côte d’Azur, CNRS, CS 34229, Nice Cedex 4, 06304 France; 13 Department of Physics and Astronomy, Michigan State University, East Lansing, 48824 MI USA; 14 UCL Mullard Space Science Laboratory, Holmbury St. Mary, Dorking, RH5 6NT UK; 15 European Space Technology Centre (ESTEC), European Space Agency, Keplerlaan 1, 2200 AG Noordwijk, The Netherlands; 16 Centre for Exoplanets and Habitability, University of Warwick, Gibbet Hill Road, Coventry, CV4 7AL UK; 17 Centre for Space Domain Awareness, University of Warwick, Gibbet Hill Road, Coventry, CV4 7AL UK

## Abstract

Small bodies exist in distinct populations within their planetary systems. These reservoir populations hold a range of compositions, which to first order are dependent on formation location relative to their star. We provide a general overview of the nature of the reservoirs that source exocomets, from the influence of the stellar environment through planetesimal formation to comparisons with Solar System populations. Once transitioned from a young protoplanetary disc to a debris disc, a star can expect to be rained with exocomets. While exocomets are predominantly detected to date at A-type stars, planetesimals plausibly exist across a range of stellar masses, based on exoplanet abundance, debris disc occurrence and white dwarf infall.

## Introduction

The small-body populations of a planetary system typically exist in discrete dynamical groups: *reservoirs*. These are remnant populations of small worlds (often for generality termed ‘bodies’ or ‘objects’) orbiting their star after the dispersal of the gas disc. As every exocomet is sourced from some point within a planetesimal disc, understanding the reservoirs of a system is necessary to develop an understanding of the potential origin, composition, and thermal history of an object that is only seen in the late brief flare of a demising exocomet.

**Fig. 1:**
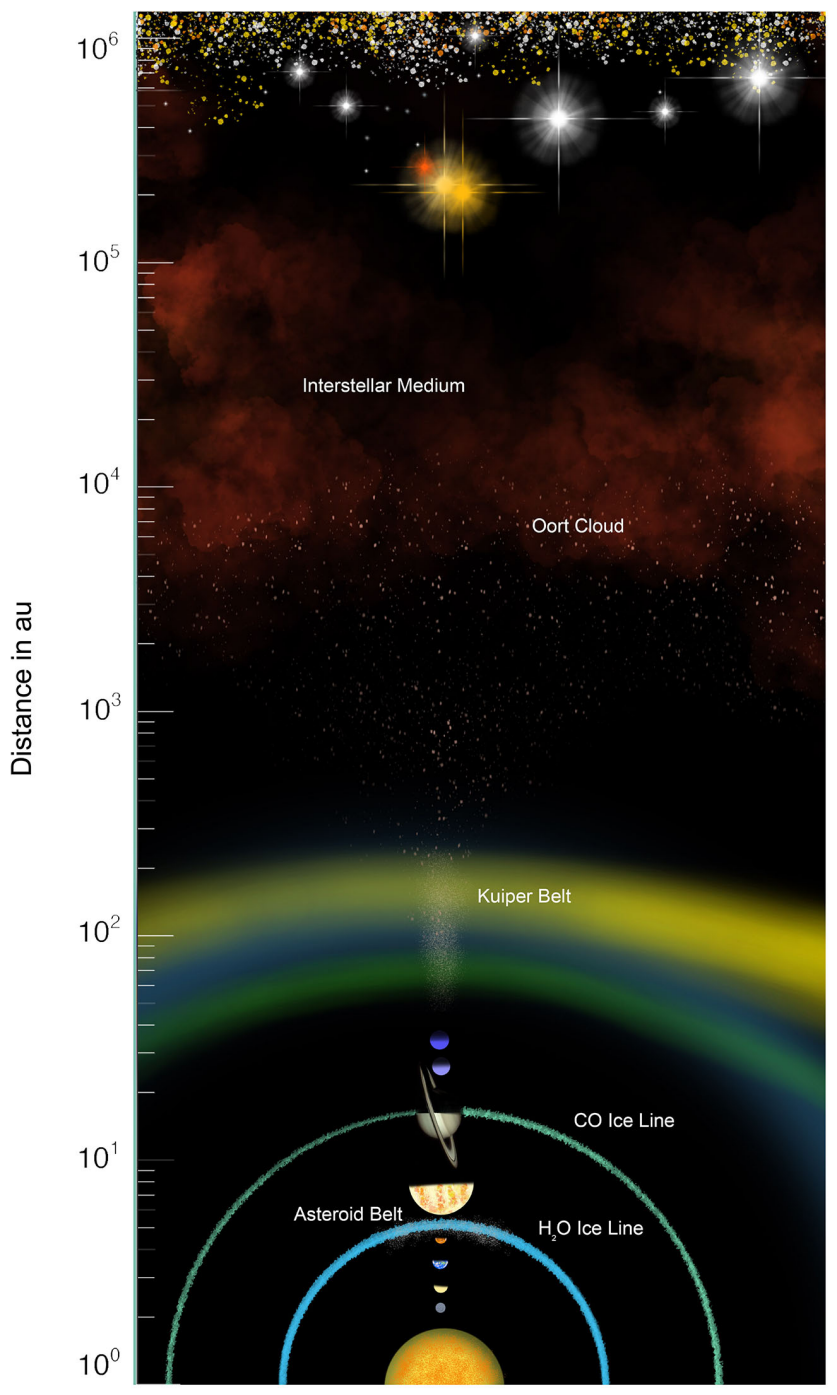
Schematic picture illustrating the approximate location of the different small-body and comet reservoirs in the Solar System

In the Solar System, observational surveys over many decades have refined our understanding of the small-body reservoirs that exist today across a wide range of semi-major axes (Fig. 1). Smallest in semimajor axis are the sparse population of ‘Ayló‘chaxnim asteroids, found entirely interior to Venus’s orbit (0.72 au) (Bottke et al. 2002; Bolin et al. 2023); no asteroids have been found within the orbit of Mercury, despite two centuries of searches for such “Vulcanoids” (Perrine 1902; Steffl et al. 2013). Other populations of near-Earth asteroids are within or cross Earth’s orbit (Atiras, Atens, Apollos and Amors respectively), with some thirty thousand greater than 100 m diameter (Nesvorný et al. 2024; Deienno et al. 2025).

The above asteroids are sourced by dynamical erosion from the larger reservoir of the main belt of asteroids with $2 \lesssim a \lesssim 3.5$ au, where $a$ is the semi-major axis, between the orbits of Mars and Jupiter. A complex structure, the main belt exhibits compositional mixing of largely volatile-poor objects rich in silicates and some organics in the inner main belt, with an overall gradient of more water-rich objects toward the outside of the belt (DeMeo and Carry 2014). The main belt is sculpted by dynamical resonances with Jupiter to leave the Kirkwood gaps. Asteroids displaying cometary or non-gravitational activity are now observationally well established in this region (Hsieh and Jewitt 2006), though only recently confirmed using JWST to be due to volatile outgassing (Kelley et al. 2023; Hsieh et al. 2025). Various co-orbital asteroids are expected (and found) for most planets from Venus outwards (Pan and Gallardo 2025), either as highly transient 1:1 resonances in horseshoe or quasi-satellite orbits, or as more permanent Trojan populations, depending on the exact orbital stability regions for each planet (Morais and Morbidelli 2006; Carruba et al. 2025; Granvik et al. 2012; Fedorets et al. 2017; Tabachnik and Evans 1999; Greenstreet et al. 2024; Alexandersen et al. 2021).^1^

At larger semimajor axes, resonant populations become more and more numerous. The Hildas are outside the main belt in a 3:2 resonance with Jupiter, numbering some six thousand, but the Jupiter Trojans are a far larger population. While no objects are currently known on stable orbits between Uranus and Neptune, they are theoretically possible (Holman 1997; Zhang and Gladman 2022), though the population must be small (of order 80 to $H_{r} < 10$;, where $H_{r}$ is the absolute magnitude, Dorsey et al. 2023). The clouds of irregular satellites orbiting each giant planet appear to originate from the same reservoir as their Trojans, and were captured at some point (Jewitt and Haghighipour 2007).

The massive population of the Neptune Trojans (Parker 2015; Lin et al. 2021) is a harbinger of the true scale of small-body populations: they are far and away the province of the outer Solar System. As the outermost known planet, Neptune’s gravitational influence sculpts the whole trans-Neptunian region; for a detailed review, see Gladman and Volk (2021). Here exterior resonances at larger semimajor axis than that of Neptune do not remove objects as in the Kirkwood gaps, but instead hold stable populations. Two main trans-Neptunian populations are present. With $42.5 < a < 47.5$ au, the cold classical Kuiper belt is a dynamically quiescent group that appears to have been retained in situ (Batygin et al. 2011; Gladman and Volk 2021). Overlapping this population are several populations, which may be grouped together as dynamically excited: objects in a wide variety of mean-motion resonances with Neptune, those in the dynamically hot classical Kuiper belt, the scattered disc that are actively changing semimajor axis due to interactions with Neptune, the fraction of scattering TNOs that are weakly sticking to distant resonances of Neptune while evolving in $a$, and more distantly in perihelia, the detached disc and the less-understood population of extreme trans-Neptunian objects. Together, these populations are both more massive and more numerous (estimated at $>10^{6}$ objects down to an absolute magnitude $H_{r}<12$; Lawler et al. 2018) than all inner populations; for a detailed population analysis see Crompvoets et al. (2022), Beaudoin et al. (2023).

Almost all trans-Neptunian objects have predominantly icy surface compositions, with a range of volatiles. The main species detected are H_2_O, CO_2_ (and its isotopologue ^13^CO_2_, CO, CH^4^, and CH^3^OH and complex organics and molecules containing aliphatic C–H, C≡N, O–H and N–H bonds (Brown 2012; Pinilla-Alonso et al. 2025). As in the inner system, a small fraction of bodies excited from the scattering disc will transition into dynamically temporary populations. These populations, the Centaurs and the short-period or Jupiter family comets, cross the orbits of inward giant planets (Fraser et al. 2022). We consider the orbital structure and compositions of the most distant and most numerous outer Solar System reservoir, the Oort cloud, in more detail later in the article. Comet surveys imply it comprises ${\sim 10^{11}}$ objects larger than ${0.3~\text{km}}$ in radius, with semimajor axes above ${\sim 1000\text{ au}}$, though these numbers are highly dependent on assumptions about the comet infall rate, flux-radius relation, and orbital distribution (Kaib and Volk 2024).

In this article, we discuss the process of crafting an exocomet, from its star’s birth environment to the formation of a planetesimal in the protoplanetary disc, and the ways this process may vary across the range of stellar environments. We describe the relationship of formation theory and Solar System analogues seen in the present day in the reservoirs in our system, used as empirical tests. As discs evolve, they move from the protoplanetary stage of planetesimal formation to a stage with collisional evolution, termed debris discs. The mature stage of our main-sequence Solar System seen today comprises several main reservoirs of bodies considered comets, such as the Oort cloud. Common dynamical processes suggest these reservoirs are likely to exist in other systems, though the level of observational evidence varies.

Once formed, reservoirs of small bodies are not static populations: they evolve with time, both through dynamical processes and through a broad range of physical processes that modify the interior and surfaces of individual bodies. These evolutionary effects are considered in Mustill et al. (2025), this collection.

## Star Formation and Its Environment

Stars are formed in dense molecular clouds, also often called dense cores, with a scale of 0.1 pc through their gravitational collapse (e.g., Myers and Benson 1983; Shu et al. 1987). Protostars deeply embedded in dense cores are optically invisible, whereas they are identified as infrared sources (e.g., Beichman et al. 1986). Some dense cores, however, have no infrared sources, and such dense cores are considered to be in the stage of pre-star formation (e.g., Bergin and Tafalla 2007). Dense cores forming stars not only gravitationally collapse but also often slowly rotate (e.g., Goodman et al. 1993), and infalling materials conserve their specific angular momenta (e.g., Ohashi et al. 1997), forming discs around central protostars (e.g., Ohashi et al. 2014). Discs around protostars are often called protostellar discs. They are also sometimes called embedded discs because they are still embedded in infalling envelopes. Discs are considered to gradually increase their masses and sizes as central protostars grow (e.g., Terebey et al. 1984), although the details of such evolution are still under debate (e.g., Sheehan et al. 2022; Yen et al. 2024). When materials in dense cores infall, a fraction of material is removed as outflows or more collimated jets, which carry away angular momentum of the infalling materials. Optically invisible protostars eventually become optically visible pre-main-sequence stars when infalling materials are consumed, both through mass infall to the central star-disc systems and also through mass outflows.

Young stars are categorized into four stages based on their evolution and surrounding matter: class 0, class I, class II, and class III (Williams and Cieza 2011). This classification relies on the slope of the spectral energy distribution (SED) in the 2–20 μm range. Class 0 stars are strongly embedded and lack complete emission in this range. Class I shows a blackbody component from the central object, with most energy from a dusty cocoon. Class II, or young T Tauri stars, primarily emit from the central object, with some contribution from an optically thick disk. In Class III, the disk is optically thin and significantly less massive.

These discs around young stellar objects are considered to be the site of planet formation. Indeed, recent direct imaging of protoplanets in the disc around T Tauri star PDS 70 (Benisty et al. 2021) strongly support this notion. In addition, recent ALMA observations at high angular resolution reveal that substructures, such as rings and gaps, are ubiquitous in discs around Class II sources (e.g., Andrews et al. 2018; Cieza et al. 2019). Although the origin of these substructures is still under debate (e.g., Okuzumi et al. 2016; Takahashi and Muto 2018), these substructures strongly suggest that planet formation actually takes place in discs around Class II sources (e.g., Dodson-Robinson and Salyk 2011; Dipierro et al. 2016). Because of the importance of discs around Class II stars for planet formation, they have been extensively observed in the past decades, allowing us to do demographics of discs. Such studies provide us with overall physical conditions of discs, such as disc mass and radius (e.g., Manara et al. 2023, see more details in the next section).

In contrast to Class II discs, discs around Class 0/I protostars are still undergoing strong development. They are much more massive than discs around Class II stars and may play an important role in planet formation. Recent studies suggest that dust masses of Class II discs are insufficient to account for the solid masses of most observed exoplanets (e.g., Manara et al. 2018), while Class 0/I discs do seem to have enough mass for exoplanets (e.g., Tychoniec et al. 2020). A recent imaging survey for Class 0/I discs at a high angular resolution revealed that they have fewer distinctive substructures in marked contrast to Class II discs, suggesting that substructures could be rapidly developed when central stars evolve from protostars to Class II sources (Ohashi et al. 2023). The survey also revealed that Class 0/I discs are more spatially thick than Class II discs (Takakuwa et al. 2024). Interestingly, their disc sizes increase as a function of the central stellar mass (Yen et al. 2024). It is still not clear whether this trend is due to disc evolution or spectral type dependence.

Most stars are born within a cluster of stars (Lada and Lada 2003), not in isolation. The star cluster environment may affect the protoplanetary disc surrounding the young stars and, therefore, also the resulting exocomet populations. The environment can affect the discs in two ways – by close stellar flybys and by intense radiation (Winter et al. 2018; Concha-Ramírez et al. 2023). Both mechanisms can lead to disc truncation and even disc destruction, thus they may reduce the amount of disc material available for planetesimal formation. How strongly the environment affects the discs depends strongly on the cluster density, which can vary over seven orders of magnitude (Pfalzner and Kaczmarek 2013).

During the early disc phases ($<5$ Myr), it depends on the cluster type whether external photo-evaporation or stellar flybys are the dominant effect on the protoplanetary discs. The radiation field is only efficient in removing discs if the cluster contains rare, massive, O and B stars. This external photo-evaporation dominates in massive long-lived clusters, where the number of stars in the clusters $N_{\mathrm{stars}}$ is large and stellar density $n$ high, ($N_{\mathrm{stars}} >$ 1000, $n >$ 1000/pc^3^) (Winter et al. 2018; Concha-Ramírez et al. 2023). By contrast, flybys dominate in low-mass short-lived clusters (Pfalzner and Govind 2021).

As external photo-evaporation only affects the gas in the disc, it is no longer efficient in the debris disc phase (debris discs contain much less detectable gas than protoplanetary discs; e.g. Kral et al. 2017). By contrast, flybys may affect discs throughout the star’s lifetime. However, close stellar flybys are most common during the early phases. The frequency of close stellar flybys differs greatly depending on the type of star cluster (Adams 2010). The underlying reason for this diversity is that star clusters can be divided into two major types – long-lived and short-lived, sometimes referred to as clusters and associations (Pfalzner 2009). While both cluster types expand by about a factor of 5–10 during the first 10 Myr of their existence, their stellar density differs by a factor of 100–1000 at any given age. Thus, close encounters can happen in both environments, but are more likely in long-lived clusters. The likelihood of a close stellar flyby also depends on the star’s mass. High-mass (A-type and above) and M-type stars are more likely to undergo a close stellar flyby than intermediate-mass stars, as high-mass stars function as gravitational foci (Vincke and Pfalzner 2018).

The environment can strongly influence the properties of the disc from which the planetesimals may form. It also may lead to matter transport within the disc, potentially affecting the chemistry within the disc. The question is what role these discs’ physical and chemical properties play in forming the planetesimals.

## Initial Chemical Composition and Physical Conditions of Protoplanetary Discs

Planetesimals, and then planets, are believed to be formed in protoplanetary discs, where the number density of dust particles is high. The dust-to-gas mass ratio in these discs is commonly much higher than the typical ISM value of 0.01. This high dust density allows the dust to grow to larger sizes through processes of collisional sticking (e.g., Nakagawa et al. 1986). Processes such as trapping in gas pressure bumps and the sedimentation of dust particles lead to locally high dust-to-gas ratios, further accelerating the formation of planetesimals. Recent ALMA-survey observations of protoplanetary discs show a large variety of dust masses ranging from $\sim 10^{3}$M_⊕_ to $\sim 0.1$M_⊕_, depending on the age of the discs (Bae et al. 2023). The uncertainty in measuring the gas mass in the disc is quite large, since neither the gas-to-dust mass ratio nor the CO-to-H_2_ gas mass ratio are uniform in the discs, which is different from the case of molecular clouds. A recently proposed method to measure the mass of the disc gas using the pressure broadening of the CO transition lines shows that a protoplanetary disc has gas of $\sim 7$ Jupiter masses inside the disc radius of $\sim 5$ au (Yoshida et al. 2022). The measured sizes of the discs are very different, depending on the observed tracers. For example, the size traced by CO transition lines or infrared dust-scattered light is more than five times larger than that traced by millimeter dust continuum emission in a protoplanetary disc, which could be explained by the drift of pebbles (mm-dm grains) towards the central star. ALMA observations can resolve the dust continuum emission, showing that the majority are compact (the disc radii of $\leq 50$ au), while some discs have larger disc radii of up to $\sim 200$ au (e.g., Miotello et al. 2023).

The composition of the reservoirs is significantly controlled by the thermal conditions. The materials in the discs are heated by stellar irradiation, and the temperature profile depends on the stellar luminosity and the distance from the central star. Turbulent viscosity could also heat the materials near the midplane of the inner discs, depending on the strength of the turbulence. In addition, the properties of the dust grains, such as the size distribution and spatial distribution, affect the temperature profile of the discs through the opacity of the dust (e.g., Oka et al. 2011; Harsono et al. 2015). Dust grains near the midplane of the outer discs are cold because they are shielded from the direct irradiation of the star, so that gas-phase species are frozen out on dust grains and subsequent surface reactions proceed until dust grains are locked into planetesimals and larger objects, the disc gases disperse, or the grains drift past a snow line.

In the hot inner discs, water and organic molecules thermally vaporise into the gas phase. The boundary outside of which a certain molecule exists as a solid is called its ‘snowline’, and the location of the snowline depends on how easily each molecule sublimates. For example, carbon monoxide (CO) sublimates at a lower temperature than water. The sublimation temperatures of CO and water are around 20 K and 150 K, respectively, with the location of the CO snowline being around 20–30 au, while the location of the water snowline is around 1–2 au in protoplanetary discs around solar-mass stars. The location depends on the luminosity of the central star, and for more luminous stars, the snowlines appear at much greater distances from the disc. Because water is the main component of ices, the composition of planetesimals (icy/rocky) created in the discs varies significantly inside and beyond the water snowline; the mass of ice is significantly reduced inside the snowline, where only refractory organic matter and water contained in hydrous minerals, such as some silicates, can exist as solids.

Recent ALMA observations and model calculations suggest that the compositions of gas and ice are not simply controlled by the molecular snowlines. Observations have revealed CO gas depletion in the outer discs as well as even inside the CO snowline (e.g., Zhang et al. 2019; Yoshida et al. 2022). The CO gas depletion is likely to proceed as the discs evolve from Class 0/I to Class II objects with a timescale of approximately one million years (e.g., Bergner et al. 2020; Zhang et al. 2020). It is explained by theoretical models through the destruction of CO in the gas-phase by UV radiation and/or cosmic rays and the subsequent freeze-out of molecules on dust grains following grain surface reactions, which is stimulated by turbulent mixing of gas and dust in the discs (e.g., Krijt et al. 2020; Furuya et al. 2022). Anomalies in elemental abundances in carbon, oxygen, and nitrogen in the gas phase in protoplanetary discs have been suggested by recent ALMA observations. The carbon-to-oxygen elemental abundance ratio is higher than one (C/O$>1$), in contradiction to the local interstellar medium and our Solar System, and even the nitrogen elemental abundance is larger than carbon and oxygen elemental abundances in gas-phase in some discs (e.g., Öberg et al. 2023; Krijt et al. 2023). Such anomalies are also explained by the above-mentioned model with freeze-out of molecules on dust grains with different tendency depending on the elements; oxygen, carbon, and nitrogen are more likely incorporated into ice in this order. The composition of icy planetesimals must depend on where and when they form in the discs.

The type of star is one of the keys to determining the location of the snowlines. The more luminous the star, the more distant the snowlines are situated. As a result, the ice lines of more massive stars are positioned at significantly greater distances than those around solar-type stars. In the next section, we explore the impact of the mass of the host star on planetesimal formation and, consequently, the creation of exocomet reservoirs. Notably, exocomets have been predominantly/exclusively discovered in the vicinity of A stars.

## Dependence on Host Star Mass

The existence of exocomets requires both a source population (a disc or Oort-like cloud) and one or more massive bodies in the system (planets or stars) to drive the cometary nuclei down to small pericentres. The formation of these populations depends on conditions of star and planet formation, which in turn depend on the mass of the primary (proto-)star. Meanwhile, our observations are differentially sensitive to bodies orbiting stars of different masses.

Observationally, we have a good understanding of stellar multiplicity as a function of stellar mass, with the fraction of stars in binaries (or higher multiples) rising with mass, from around 1 in 4 for M dwarfs, 1 in 2 for Sun-like stars, to over 60% for B stars (Duchêne and Kraus 2013). Detection of lower-mass (planetary) companions becomes strongly affected by detection biases for many regions of parameter space, while precise occurrence rates for a particular class of planet (such as “Jupiter-like”) can be sensitive to the cuts in semimajor axis (or period) and planetary mass $m$ (or $m\sin I$, where $I$ is inclination). We content ourselves here, therefore, with a reasonably qualitative description: Exoplanet demographics is a complex field, and many reviews exist (e.g., Zhu and Dong 2021).

In the context of exocomets, we are primarily interested in wide-orbit planets beyond a few au, where planets are close to the source reservoir. Here, the most prolific detection method (planetary transit) fails. Direct imaging is most powerful at wide separations, albeit only to gas-giant (Jovian or super-Jovian) planets at present. Roughly 10% of B stars host such planets detectable by direct imaging (Janson et al. 2021; Delorme et al. 2024). This is roughly comparable to the occurrence rate of wide-orbit giant planets around Sun-like stars, although other imaging surveys reveal a positive correlation with stellar mass (Nielsen et al. 2019; Vigan et al. 2021). White dwarf studies show that about 10% of white dwarfs whose main-sequence progenitor masses were above $3.5\mathrm{\,M_{\odot }}$ currently host, and therefore once formed, planetary systems (Ould Rouis et al. 2024). One of the highest of these masses is $4.8\mathrm{\,M_{\odot }}$, for the white dwarf WD J2317+1830 (Hollands et al. 2021). Radial-velocity surveys again reveal roughly similar rates, with a peak in giant planet occurrence at around $2\mathrm{\,M}_{\odot}$ (Reffert et al. 2015); note that this relies on study of *evolved* former A stars, as main-sequence A stars have too broad spectral lines for precision RV measurements. For reasons discussed in Mustill et al. (2025), this collection, lower-mass planets may be preferred as dynamical drivers of exocometary activity. Unfortunately, sub-Jupiter planets on wide orbits are essentially invisible with current technology. However, some are picked up in gravitational microlensing surveys, which suggest occurrence rates of at least tens of per cent for planets roughly Neptune mass or larger located around or beyond the snowline (Shvartzvald et al. 2016; Poleski et al. 2021). The host stars probed by these surveys tend to be low mass (M dwarfs). The overall picture, then, is a positive correlation between stellar mass and the presence of a wide-orbit Jupiter-like planet, at least up to $\sim 2\mathrm{\,M}_{\odot}$, while lower-mass planets are considerably more numerous but too hard to probe to yet say whether their presence on wide orbits depends on stellar mass.

The occurrence rates of close-in planets ($\lesssim 1$ au) have been provided by transit surveys (e.g., Dressing and Charbonneau 2013; Mulders et al. 2015; Hardegree-Ullman et al. 2019; Yang et al. 2020; He et al. 2021), which have relatively simple biases, large sample sizes, and sensitivity to small (down to Earth-sized) planets. There appears to be a decrease in close-in planet occurrence rate between M and F-type stars, although it is unclear how this can be extrapolated to the wider-orbit planets presumably responsible for driving exocomet dynamics. These observations have motivated many studies of the dependence of planet formation as a function of stellar mass for roughly Solar-mass stars and smaller (e.g., Liu et al. 2019; Burn et al. 2021; Mulders et al. 2021). Less interest has attached to A stars, possibly because, by virtue of their smaller number, they are less common in transit surveys. Some work here has been motivated by observations of post-main sequence planetary systems: see Veras et al. (2020), Kunitomo et al. (2021), Johnston et al. (2024).

The observed prevalence of planet formation around stars of different masses indicates that exocomets should not be formed exclusively, or even predominantly, around A-type stars, where their detection is favoured (see Korth et al., this journal, for discussion). Debris discs are probably the primary source of exocomets and debris discs are more commonly detected around younger stars and, for stars of a similar age, earlier spectral types (Fig. 4, and e.g., Su et al. 2006; Krivov 2010; Wyatt 2008; Matthews et al. 2014; Moór et al. 2016; Hughes et al. 2018; Sibthorpe et al. 2018; Marino 2023; Pearce 2026). Note, however, that it is easier to detect a disc of a given temperature and fractional luminosity around earlier-type stars, as the disc emission is more clearly separated from the stellar blackbody (e.g., Wyatt 2008). In reality, all main-sequence stars probably have some amount of debris, and thus potentially exocomets. The phenomenon of exozodiacal dust within $\sim 1$ au of the host star, detectable as extended near- or mid-IR emission via interferometric observations (Ertel et al. 2014, 2020), may be related to cometary activity (Sezestre et al. 2019; Pearce et al. 2022b; though also see Pearce et al. 2020). It correlates strongly with the presence of a detected cold debris disc in the system, and detected presence declines somewhat with lower stellar mass (comparing A–early F with late F–GK stars), but the latter can be explained by increased detection sensitivity for earlier spectral types (Ertel et al. 2020).

## Early Stages of Planetesimal Formation

**Fig. 2:**
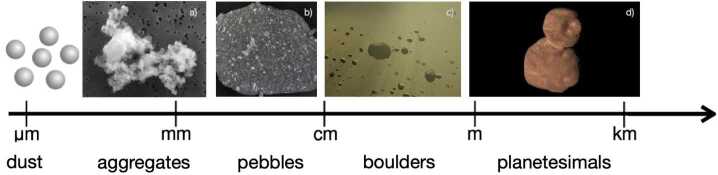
Schematic picture illustrating the growth from dust to planetesimal sizes. ‘Dust’ of silicate- and organics-rich grains is shown schematically; these grow to fractal dust structures via sticking collisions. After compactifying and further growth, they reach planetesimal sizes. Credits: a) Amara/Wikipedia (CC), b) via Wikimedia Commons, c) adapted from Wikipedia Commons, and d) cold classical Arrokoth (NASA)

It is generally assumed that dust in protoplanetary discs grows from initial sizes of ∼ 0.1 – 1 μm dust grains to planetesimals of sizes in the range ∼1–1000 km, a process shown schematically in Fig. 2. Mutual collisions among protoplanetary dust particles are important for understanding the buildup of these larger bodies. Pioneering theoretical work on dust growth (Weidenschilling 1977; Weidenschilling and Cuzzi 1993) was followed in the last two decades by complementary laboratory experiments (for a review, see Blum 2018). For dust grains or small aggregates smaller than $\sim 10-100~\upmu \text{m}$, the collisions are mainly driven by Brownian motion (Kempf et al. 1999; Dominik et al. 2007), with typical velocities around 1 mm/s. Larger aggregates (> 10-100 μm) experience a systematic drift relative to the gas, due to the sub-Keplerian motion of the pressure-supported gas. The result is a net inward force on the dust, leading to an increased radial drift velocity with larger aggregate size, peaking at around 60 m/s for 1 m diameter bodies; this limits their lifetimes to merely ∼ years at 1 au (Blum 2018).

Differences in how dust and gas stick together cause variations in the speed of drift among aggregates. Systematic drift of dust particles relative to the gas also occurs, due to sedimentary motion towards the disc midplane. Larger dust aggregates have a greater surface area-to-mass ratio. Thus, they sediment faster than smaller aggregates, resulting in collisions among aggregates of different sizes. Additionally, gas turbulence significantly influences dust aggregate evolution by causing collisions, especially among larger aggregates — leading to stochastic particle motion, regardless of their aerodynamic properties.

A key problem in aggregate growth was identified experimentally: when dust aggregates of μm- or mm-sized silicate grains collide, they bounce or fragment, rather than sticking (Blum and Münch 1993). Since this early work, the parameter space has been expanded to dust sizes between ∼ 1 μm and 10 cm, impact velocities between $\sim 10^{-3}$ m/s and $\sim 100\text{ m}/\text{s}$, and agglomerates of water ice (Gundlach and Blum 2015), CO_2_ ice and CO_2_–H_2_O ice mixtures (Musiolik et al. 2016) have been tested. Monte-Carlo simulations of the aggregation process of silicate dust grains show that after an initial process of fractal growth, sticking and bouncing collisions lead to the formation of compact mm- to cm-sized aggregates, which have relatively low volume filling factors of $\sim 0.36$ (Zsom et al. 2010). The bouncing barrier inhibits further growth, as no additional mass transfer can be achieved (Wurm and Teiser 2021). Such a bouncing barrier may not exist for icy particles (see, for example, Okuzumi et al. 2012).

Aggregate growth has some dependence on both location in the protoplanetary disc, and the disc parameter termed dust-to-ice ratio: the ratio of silicate- and organics-rich particles to those of volatile ices. The maximum aggregate size that can form at a given point in the disc depends on both the distance to the star and the composition of the dust (Lorek et al. 2018). Increasing distances to the star and dust-to-ice ratios result in smaller maximum aggregate sizes.

After reaching the bouncing barrier (Dominik and Dullemond 2024), the gap to fragmentation, mass transfer or cratering may be overcome, due to the velocity distribution of the dust aggregates. The destructive processes eroding aggregates will form new, smaller aggregates. Once these projectile aggregates develop, their mass transfer allows some dust aggregates to grow to planetesimal sizes (Windmark et al. 2012).

Although the time scales for growth to the 100-metre level are reasonably short at distances of 1 au, they are much longer further out in the disc, and the maximum aggregate sizes are thus considerably smaller (Birnstiel 2024). Garaud et al. (2013) showed that at 30 au, even after 6 × 10^5^ years, the maximum aggregate size is only a few metres. Typically, the growth time scales are so long that outside dust traps in the disc, where the radial pressure gradient of the gas vanishes, halting dust drift, radial drift limits the maximum size achievable. The formation of planetesimals likely proceeds through alternative pathways, as described in the next Section.

## Late Stages of Planetesimal Formation

### The Challenge of Planetesimal Formation

Planetesimals form most likely through the gravitational collapse of clouds of pebbles (mm–dm grains) where these can locally accumulate in the disc, reaching densities exceeding the Hill density where self-gravity overcomes the Keplerian shear that tears the cloud apart. The process was initially described in the foundational work by Safronov (1972) and Goldreich and Ward (1973). This original idea was then largely abandoned for several decades, as Weidenschilling (1980) pointed out that at the necessary high dust-to-gas ratios for gravitational collapse in the midplane, strong turbulence will be triggered by vertical shear in the disc. That is, the dust-dominated midplane will rotate at a Keplerian value, whereas the gas-dominated layers above and below will rotate at sub-Keplerian value, due to the radial pressure gradient in the gas. This results in the development of the Kelvin–Helmholtz Instability (MRI; Chandrasekhar 1961), and so the dust concentration should be quickly diffused by turbulence, before a gravitational collapse can happen.

The conclusion by Weidenschilling (1980) was that possibly coagulation, i.e. non-elastic and sticking collisions of dust grains, leads to growth up to the sizes of comets and asteroids. Yet as discussed in Sect. 5, extended experimental data on collision velocities that would lead to bouncing and fragmentation rather than sticking showed that this pathway to planetesimal formation is not possible (Blum 2018).

Much attention has therefore focused on identifying conditions that might permit sufficiently dense clouds of pebbles to accumulate and collapse. An early suggestion was concentration by Kolmogorov turbulence in the gas disc: particles can stochastically be driven to high densities between turbulent eddies (Cuzzi et al. 1993; Pan et al. 2011; Hartlep and Cuzzi 2020). However, the stochastic nature and short lifetime of these eddies mean that the probability of attaining the high density required may be low.

Many disc processes, however, give rise to large-scale structures that can trap particles. Particle trapping is systematic in slowly-rotating, long-lived vortices (Barge and Sommeria 1995; Klahr and Henning 1997), which do not appear in the Kolmogorov turbulence invoked in Cuzzi et al. (1993). However, in both hydrodynamical (Raettig et al. 2015; Manger and Klahr 2018) and magnetohydrodynamical simulations (Johansen and Klahr 2005; Johansen et al. 2006b; Lesur et al. 2023), the formation of such particle-trapping vortices, as well as pressure bumps, is a common phenomenon. These can be driven, for example, by the magnetorotational instability (MRI; Balbus and Hawley 1991), and early simulations of turbulent discs driven by the MRI and including the self-gravity of pebbles showed rapid formation of planetesimals in zonal flows (Johansen et al. 2007).

Particle concentration is aided by vertical settling, which Weidenschilling (1980) argued should be prevented by KHI. However, the first direct numerical simulations of the KHI (Johansen et al. 2006a) of pebbles sedimenting to the midplane of the disc showed that while on average pebbles diffused away from the midplane, strong local clustering in fact occurred. Such simulations of turbulence caused by sedimentation of particles have often been interpreted as the streaming instability, discussed next.

### The Streaming Instability

**Fig. 3:**
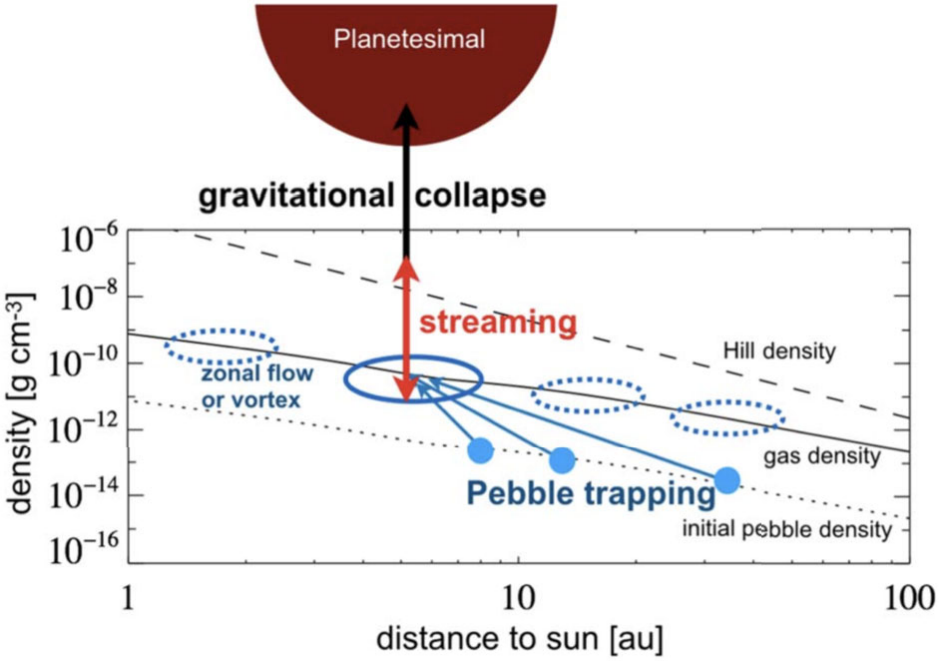
Drift-trap-stream-collapse (schematic representation of densities). In the current paradigm the sequence of planetesimal formation starts with particles growing to pebble size; they then sediment to the midplane and drift toward the star to get temporarily trapped in a zonal flow or vortex (Lenz et al. 2019a). Here the density of pebbles in the midplane is eventually sufficient to trigger the streaming instability. This instability concentrates and diffuses pebbles likewise, leading to clumps that reach the Hill density at which tidal forces from the star can no longer shear the clumps away. If the turbulent diffusion is now also weak enough to let the pebble cloud collapse, planetesimal formation will occur.

The streaming instability (SI) arises as a result of the back-reaction of the pebbles on the gas: pebbles orbit at the Keplerian velocity, while the gas disc’s azimuthal velocity is affected by the radial pressure gradient. The drag force on the solids also causes the gas to be accelerated towards the solid velocity, which process becomes more significant as the solid-to-gas ratio increases. Particles embedded in the gas drift radially inward due to their lack of pressure support to balance the diminished centrifugal force caused by the sub-Keplerian gas motion. This drift results in local accumulation of solid particles (see Fig. 3), as follows.

The streaming instability relies on a fundamental oscillation mode of the disc gas, in which the gas moves diagonally (radial–vertical) while conserving its angular momentum. This is a generalization of the familiar epicyclic oscillations of particles around a circular orbit. In the part of the gas bands where the gas is at its furthest outward position during the oscillation, the gas has lower angular momentum than its surroundings. As a result, the particles drift faster than average, while at the opposite point in the oscillation cycle, the particles drift slower. Consequently, the particles accumulate at the neutral point of the oscillation, when the gas moves outward, giving the gas a “kick” towards the Keplerian value. In contrast, the inward-moving gas experiences a deficiency of dust, and this results in an inward “kick” on the gas. If the particles in the gas move radially inward at the same velocity as the phase velocity of the gas oscillations, a resonant amplification of the gas oscillation occurs (from this, SI can also be classified as a resonant drag instability; Squire and Hopkins 2018). As a result, SI can amplify the local dust-to-gas ratio by an order of magnitude before nonlinear effects kick in. As the radial drift velocity of the pebbles depends on their stopping time, the wavelength, oscillation frequency and thus the phase velocity of the amplified oscillation are strongly dependent on the particle size (Youdin and Goodman 2005). This has been argued as an obstacle for SI if one has a wide spectrum of particle sizes (Krapp et al. 2019), though problems may not arise at sufficiently high dust-to-gas ratios (Schaffer et al. 2021; Zhu and Yang 2021; Yang and Zhu 2021).

The streaming instability relies on a significant back-reaction from the dust on the gas, thus requiring a sufficiently high dust-to-gas ratio. The initial condition to trigger SI is a local dust-to-gas ratio of at least order unity, similar to the condition for KHI. This can be attained through vertical sedimentation; however, a super-Solar overall metallicity (about 2–3 times Solar) is required to overcome turbulent diffusion and trigger gravitational collapse (Johansen et al. 2009; Carrera et al. 2017; Yang et al. 2017; Gerbig et al. 2020; Klahr and Schreiber 2020, 2021; Li and Youdin 2021; Lim et al. 2024). The required high levels of solid content can be potentially caused by photo-evaporation in late-stage discs or early trapping in vortices and zonal flows (Carrera et al. 2017; Lenz et al. 2019b). The key condition for planetesimal formation may not merely be reaching a midplane dust-to-gas ratio of unity, but achieving sufficient local mass to reach Hill density. Overcoming this threshold minimizes turbulence effects and facilitates gravitational collapse (Johansen et al. 2007).

It has now become common to refer to the simulations of turbulence by sedimenting pebbles as ‘planetesimal formation via the streaming instability’. However, we want to stress that the role of SI in the generation of the high pebble-to-gas ratios, beyond the typical one order of magnitude, has not yet been explained (Li et al. 2018). Yet for the conclusions from the related numerical work with and without the inclusion of extra turbulence by other mechanisms than the particle feedback, it is not relevant to check for the role of SI. It may become important in the future once we better know parameters such as the actual pebble size distribution, the actual level of turbulence in the gas nebula, and finally, its global mass content of solid material.

### Initial Mass Distribution

Simulations of planetesimal formation in the so-called ‘SI scenario’ reveal size distributions with shallow power laws, with mass distributions such as $dN/dM_{\mathrm{P}} \propto M_{\mathrm{P}}^{-p}$ ($p \approx 1.6$ or $p \approx 1.3$ for truncated distributions) favoring large planetesimals (Johansen et al. 2015; Simon et al. 2016; Schäfer et al. 2017; Abod et al. 2019; Ormel and Huang 2025). High-resolution studies suggest a critical collapse mass may explain the deficiency of smaller planetesimals, emphasizing the role of turbulent diffusivity (Klahr and Schreiber 2021). The initial size of planetesimals depends on the size distribution of pebbles and the level of turbulence in the gas nebula.

Many planetesimals from the initial disc merged into planets, but the remnant populations, such as asteroids and trans-Neptunian objects, and in other systems exo-comets, are diagnostic of the original populations. Studying these bodies provides key insights into planetesimal formation and planetary evolution, though reconstructing their initial size distribution requires accounting for the system’s lifetime of collisional evolution.

In the Solar System, when using the parameters of Lenz et al. (2020), one finds that both asteroids and cold classical KBOs should form with a diameter of $\approx 100\text{ km}$. Observations reveal that some asteroids, predominantly ∼100 km in size, are not collisional fragments but primordial objects, supporting this “born big” hypothesis (Morbidelli et al. 2009; Delbo’ et al. 2017). Additional evidence includes the bi-lobed structure of Arrokoth and the high binary fraction among cold classical TNOs, consistent with formation via gravitational collapse (Stern et al. 2019; Nesvorný et al. 2019). Numerical simulations of gas and dust that trace the gravitational collapse in streaming instability have demonstrated that it provides a qualitative agreement with the observed distributions of the orbital inclinations of Kuiper Belt binaries (Nesvorný et al. 2019). The observed heterogeneity of the reflectance spectra, colours, and surface composition of trans-Neptunian binaries further supports formation via gravitational collapse of a pebble concentration (Marsset et al. 2020), as does the size-dependent bulk density of TNOs (Brown 2013; Wahlberg Jansson and Johansen 2014).

How may planetesimal formation proceed around other stars? Under the assumption that the formation of exo-comets happens analogously to the formation of minor bodies in the Solar System, we can translate the predicted mass for planetesimals $m_{c}$ from that for Solar-type stars. Klahr and Schreiber (2021) predict $m_{c}\propto M$, where $M$ is the stellar mass. Therefore, the radius of these icy and rocky objects will not depend strongly on the stellar type. As in the Solar System, planetesimals will obtain typical diameters of about 100 km at the time of their formation. The size may also depend on formation location in the disc, with a mass predicted to increase roughly linearly with distance from the star (Li et al. 2019; Liu et al. 2020).

## From Protoplanetary Disc to Debris Disc

Looking now at the entire extent of a protoplanetary disc, the dust growth, pebble and planetesimal formation happen with different efficiencies, and may not be simultaneous everywhere in the disc. While early dust growth is observable, the planetesimal growth process itself is not directly observable. However, we can consider how long it takes for protoplanetary discs to turn into debris discs. The lifetime of a protoplanetary disc is defined as the time it takes to disperse most of its gas. At this point, gas giants can no longer be formed. Initially, it was assumed that by 6 Myr, all stars would have lost their discs (Haisch et al. 2001), with typical disc half-life times of 1–3 Myr. However, it turned out that such short disc lifetimes are only characteristic of stars of at least 0.8 solar masses. The lifetime of protoplanetary discs is a strong function of the mass/stellar type of the host star (Ribas et al. 2015). A-type stars, where exocomets have most typically been observed to date, typically have relatively short proto-planetary disc lifetimes (1-4 Myr), while the smaller M- and K-type stars have mean disc lifetimes of 5–10 Myr (Michel et al. 2021; Pfalzner et al. 2022). The distribution of the protoplanetary disc lifetime is very broad, so that some M-type stars can still be surrounded by a protoplanetary disc at an age of 20 Myr, with some rare examples still having a disc at 40 Myr and older (e.g., Silverberg et al. 2020).

Once the protoplanetary disc disperses, we are left with a disc of remnant solid material that did not make it into planets. This is the debris disc. A sizeable potential reservoir of exocomets in nearby stars is their cold debris discs, which are located in the outer regions of systems and are detected around 20% of nearby AFGK stars (Wyatt 2008; Hughes et al. 2018). These dusty discs are brighter analogues of the Solar System’s classical Kuiper belt, and are thought to have compositions similar to those of TNOs (i.e. rich in water and hypervolatiles; Matrà et al. 2017). These discs are found to be very diverse, some of them being as narrow as the present-day classical Kuiper belt (cf. 30–50 au, though at different radii) and a majority being much wider and extending beyond 100 au (Marino 2023; Pearce 2026; Matrà et al. 2025). Some show morphological features such as warps, gaps, eccentric shapes, clumps and sharp edges (Golimowski et al. 2006; Marino et al. 2018; Faramaz et al. 2019; Booth et al. 2023; Imaz Blanco et al. 2023); these may be indicative of planetary interactions, which could drive material into the inner system to become exocomets (e.g. Mouillet et al. 1997; Pearce and Wyatt 2014, 2015; Sefilian et al. 2021; Friebe et al. 2022; Booth et al. 2023; Pearce et al. 2024). The specific dynamical mechanisms by which material could be driven into the inner system are described in Mustill et al. (2025), this collection.

How massive these discs are is an open question. Observations in the infrared and millimetre wavelengths are only sensitive to the μm- to cm-sized grains, which are most likely a small percentage of the total disc mass. These observations constrain the dust disc masses (up to cm-sized grains) to $10^{-3}-1$ $M_{\oplus}$ (Matrà et al. 2025), the lower end being limited by the current sensitivity of observatories. We expect much larger bodies to be present, whose collisions replenish the observed dust levels in a collisional cascade. If we extrapolate their dust masses to total mass assuming the largest exocomets/planetesimals are 1 km in size and a size distribution exponent of −3.5, we expect these discs to have masses of 0.3-300 $M_{ \oplus}$. It is possible that there are even larger bodies present, in which case disc masses could be even larger, but it is very unlikely that disc masses are larger than ∼1000 $M_{\oplus}$ given the available solid mass in protoplanetary discs (Krivov and Wyatt 2021). However, since the dust masses observed in many extrasolar debris discs are considerably greater than in our own Solar System (e.g. Pearce 2026), it is plausible that there is significantly more material in those exocometary reservoirs than in our own.

**Fig. 4:**
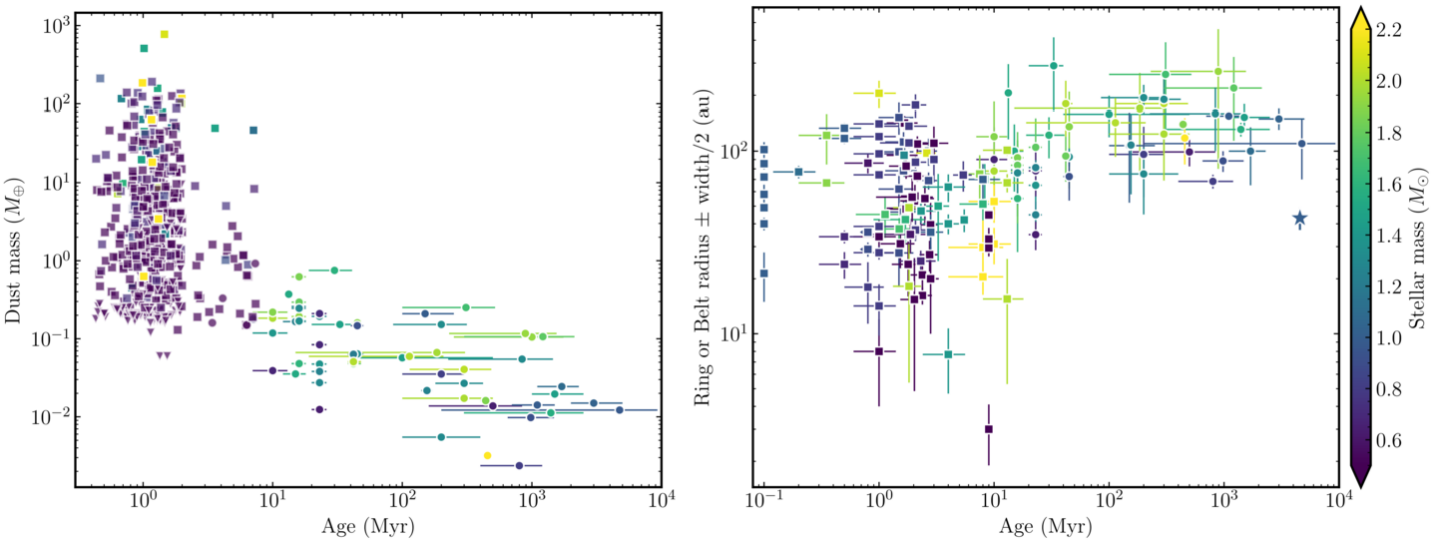
Dust mass (left) and radius (right) of protoplanetary and debris discs as a function of system age. Protoplanetary discs are represented with squares and debris or evolved discs with circles. The Kuiper belt is represented with a star on the right panel. Debris data from Matrà et al. (2025), and protoplanetary disc data from Bae et al. (2023). The colour scale indicates the stellar mass in both panels

The material in debris discs depletes over time, as the bodies grind down through mutual collisions. The resulting dust, when small enough, is removed from the system via radiation forces (see Mustill et al. 2025, this collection). This is evidenced by dust mass decreasing with age in observed debris discs, as shown on the left panel of Fig. 4. This means that older debris discs have less material available to become comets than younger ones, so we may expect exocomets to be preferentially detected in younger systems.

This collisional grinding implies that planetesimals in debris discs have non-zero eccentricities and/or inclinations, to enable frequent and violent collisions that destroy bodies to release dust (Wyatt 2008). There is some observational evidence of this; ALMA observations show that some debris discs have smooth edges, as expected from a population of eccentric bodies (Marino 2021; Imaz Blanco et al. 2023; Rafikov 2023), as opposed to the sharp edges expected if discs are sculpted by planets (Pearce et al. 2024). However, it is thought that planetesimals form in protoplanetary discs on near-circular orbits, then some processes excite them up to moderate eccentricities to enable destructive collisions in debris discs. Similarly, since debris discs are the presumed reservoirs of (exo)comets, some process must change the orbits of some planetesimals significantly to drive them onto highly eccentric cometary orbits. There are several mechanisms that can do this, as described in Mustill et al. (2025), this collection.

## Observational Evidence from the Solar System

In both the Solar System and beyond, we can learn about a system’s formation by studying extant planetesimal populations or debris discs. The locations of such discs tell us where planetesimal formation succeeded, but planet formation failed. This could be due to planet-formation timescales being too long in the outer region of systems, or because already formed planets perturb nearby planetesimals and prevent further formation (e.g. Petit et al. 2001; Eriksson et al. 2021), or because newly formed planets create pressure bumps in the protoplanetary disc, which can facilitate planetesimal formation (e.g. Miller et al. 2021). Furthermore, debris discs contain clues about where planets are and how they migrated; the past migration of Neptune is imprinted on the orbits of resonant trans-Neptunian objects (Malhotra 1993), and the shapes, features and locations of extrasolar debris discs may be telling us about planetary interactions in those systems too (e.g. Mouillet et al. 1997; Sefilian et al. 2021; Friebe et al. 2022; Pearce et al. 2022a; Booth et al. 2023; Pearce et al. 2024).

While exocomets and debris discs around other stars have only been studied for a few decades, the small-body populations in the Solar System have been the subject of astrophysical and spacecraft exploration for much longer. Alongside remote and in-situ observations, laboratory sample investigations have also greatly contributed to our understanding of the composition and material properties of not only meteorites, but also of samples from asteroids by Hayabusa (Tsuchiyama et al. 2011), Hayabusa2 (Oba et al. 2023), OSIRIS-REx (Lauretta et al. 2024) and comet Wild 2 by Stardust (Brownlee et al. 2006). One of the main drivers of these research efforts has been to constrain the processes of planetesimal formation across the Solar System’s protoplanetary disc, as well as the subsequent early dynamical evolution, which has shaped the architecture of the Solar System. However, one fundamental challenge in using the properties of the current small bodies as evidence for the early stages of the Solar System is these bodies’ subsequent evolution. The small-body populations we observe in the Solar System today have undergone various levels of dynamical, physical and chemical processing (see Mustill et al. 2025, this collection). Therefore, linking the observed properties of today’s populations to their early progenitors relies on a careful consideration of the variety of processes that have affected them since formation.

With this caveat in mind, in this section, we highlight some of the observational evidence that has been used to address key questions about Solar System formation. These examples are selected to illustrate the diversity of research questions, lines of evidence, and approaches currently being pursued by Solar System observers.

Of all available observables, the orbital distribution of the populations is probably the most widely accessible property. The orbits and relative populations of the main small-body reservoirs and their subpopulations are becoming well characterized (e.g. see Jedicke et al. (2015) for asteroids and Gladman et al. (2008), Kavelaars et al. (2020) for TNOs), with the exception of the Oort Cloud, which retains substantial uncertainties (see Sect. 9). The orbital distribution of TNOs is especially diagnostic of the late disc evolution in the Solar System. The dynamical properties of the objects in the trans-Neptunian region, the irregular satellites of the giant planets and the Trojan populations are used to constrain the dynamical evolution of the early Solar System (e.g. Nesvorný 2018; Pirani et al. 2019). They are also used to quantify the influence of the stellar environment through the effects of close stellar encounters (Nesvorný et al. 2023; Pfalzner et al. 2024a,c).

The detailed characterization of the dynamical links between the outer Solar System populations reveals that comets, TNOs, irregular satellites and Trojans share a common origin in the primordial distant disc (see Nesvorný 2018; Fraser et al. 2022). All of these populations can therefore be used to discern the evolutionary properties from the primordial signatures of the conditions in their original reservoir in the outer regions of the protoplanetary disc. While faint, it is easiest to study these distant bodies’ surface properties, and they have been explored for evidence of differences in composition. Multiband photometric studies have provided strong evidence that the surface colours of small TNOs, Centaurs, Jupiter Trojans and possibly Neptune Trojans are bimodal (e.g., Szabó et al. 2007; Peixinho et al. 2015; Tegler et al. 2016; Wong and Brown 2017; Marsset et al. 2019; Fraser et al. 2023; Markwardt et al. 2023, and references therein). While the details of the different color classifications are outside the scope of this article, we want to highlight the possibility of using them as probes of the primordial planetesimal disc composition and its dependence on heliocentric distance. For example, Wong and Brown (2016) hypothesized that the different surface colors of Jupiter Trojans reflect the formation region of the objects with respect to the region in the primordial disc where H_2_S could be retained. Additionally, studies of the surface colors in combination with the dynamical properties of TNOs (e.g. Nesvorný et al. 2020; Marsset et al. 2023) have been used to probe for a compositional gradient in the primordial disc and sublimation-driven surface depletion in the early Solar System, which leads to today’s differences in surface colors. The launch of JWST has enabled us to obtain near-IR spectra of distant objects with unprecedented sensitivity. The first JWST programs are already beginning to reveal significant differences in the surface composition among the populations (e.g Licandro et al. 2025; Belyakov et al. 2024; Wong et al. 2024; Harrington Pinto et al. 2023; De Prá et al. 2025). The first large JWST program targeting medium-sized TNOs (DiSCo-TNOs) identified three distinct spectral groups among TNOs and Centaurs, giving rise to the hypothesis that the objects from these populations were formed in different locations with respect to the sublimation radius of CO_2_ and several other molecules such as CH_3_OH, C_2_H_6_ and HCN (Pinilla-Alonso et al. 2025).

Before JWST, most of the progress on compositional studies of small bodies came from active objects. Comets and active Centaurs experience outgassing, which allows us to probe the composition of their subsurface, less-altered material.

Ongoing observational surveys aim to connect the chemical abundances of comets to their formation and thus to the properties of the protoplanetary disc (Eistrup et al. 2019). These include studies of easily detectable fragment species such as OH, CN, C_2_, C_3_, and NH in hundreds of comets (A’Hearn et al. 1995; Cochran et al. 2012; Fink 2009; Schleicher et al. 2007); analyses of multiple minor species directly released from comet nuclei for 30 comets (Mumma and Charnley 2011; Russo et al. 2016; Saki et al. 2024); and measurements of the relative abundance of the main ices in a sample of 25 comets and Centaurs, i.e. H_2_O, CO_2_, and CO (A’Hearn et al. 2012; Harrington Pinto et al. 2022). However, limitations in the sample size and observational biases towards bright Oort Cloud comets in the latter two datasets make it difficult to connect these. Additionally, the chemical origins of CN and C_2_ are not understood (Feldman et al. 2004), which hampers connecting the largest dataset to the other two, or to Solar System formation. In addition, most observations capture only brief snapshots of cometary activity. Long-term studies suggest that the composition of the coma can vary significantly due to evolutionary and seasonal effects (Combi et al. 2020; Bodewits et al. 2014).

Without doubt, in-situ missions provide the most detailed compositional analysis. For example, the JAXA Hayabusa2 mission recently returned samples from the carbonaceous asteroid (162173) Ryugu that showed organic molecules (Naraoka et al. 2023; Oba et al. 2023), in contrast to the samples returned earlier from the volatile depleted stony asteroid (25143) Itokawa, which were organic poor (Tsuchiyama et al. 2011).^2^ Similarly, the Rosetta mission, and more specifically its Rosina instrument, provided a detailed inventory of the volatiles in the coma of comet 67P/Churyumov–Gerasimenko (Le Roy et al. 2015). One of the most surprising findings was the elevated D/H isotope ratio of 67P with respect to other Jupiter-family comets (Altwegg et al. 2015); the D/H ratio is believed to be a strong clue to formation location (see Jacquet and Robert 2013). However, results from the past decade reveal that interpreting the measured D/H ratio remains challenging. It cannot be ruled out that the D/H ratio estimates for a single comet vary along its orbit (Paganini et al. 2017), while the activity level of a comet seems to be linked to the measured isotope ratio (Lis et al. 2019), raising the possibility that the activity mechanisms releasing the observed gas can result in differences in the observed D/H isotope ratio. Even the ROSINA results have been challenged by the reanalysis of the archival data by Mandt et al. (2024), which identify a much lower D/H ratio than originally derived from the original Rosetta results. The rich datasets from Rosetta still reveal new key evidence. Marschall et al. (2025) were able to constrain the approximate temperature and location in the disc where comet 67P has formed, estimated to lie around 25–35 au. Other ratios also encode information about formation location and environment; for example, the volatile carbon-to-oxygen ratio can provide a zeroth-order insight into formation distance (Seligman et al. 2022).

## Oort Clouds

In the Solar System, the Oort cloud is the source of the long-period comets. When looking at reservoirs for the production of exocomets, we have so far considered the formation of the closer reservoirs and the general state of debris disc structures. Here, we look more closely at the formation mechanisms of the Oort cloud and their likelihood for other stars. For a detailed review, see Kaib and Volk (2024). There are two regions distinguished in the Oort cloud — the inner Oort cloud is located between 2000 and 10^4^ au, beyond which the outer Oort cloud starts. While not directly observable, models predict the inner cloud to be the much denser of the two, having tens or hundreds of times as many cometary nuclei as the outer cloud. Simulations of the ejection of planetesimals from the vicinity of stars show that a fraction of ∼ 1–3% of the ejected planetesimals remain bound to their parent star. The orbits of these planetesimals isotropize and circularize due to the Galactic tidal field, and eventually form an Oort cloud between $\sim 10^{4}$ and $\sim 2\times 10^{5}$ au (Portegies Zwart et al. 2021).

While long-period comets are routinely detected by sky surveys such as PanSTARRS (e.g. Boe et al. 2019), the Solar System’s Oort cloud has not been observed in situ. The existence of a distant cloud of cometary objects that orbit the Sun is based on a spike in the reciprocal orbital separation at $1/a_{0}\leq 10^{-4}$ au^−1^ (Oort 1950), where $a_{0}$ is the original semi-major axis. One promising approach for detecting Oort cloud objects in situ is by searching for their sub-second occultations of distant stars (Schlichting et al. 2012). Using ≈1 s integration by >1 m telescopes at the optimal region near the quadrature points will be marginally dominated by Oort cloud objects rather than Kuiper belt objects (Ofek et al. 2024).

The formation timing of our Oort cloud is somewhat open: it may have formed together with the Solar System, potentially during a giant-planet instability, or it might have been acquired in part later on, from other stars in the Sun’s birth cluster. It remains also an open question to what extent the Oort cloud is still in its primordial dynamical state. If the Oort cloud is a pristine relic of Solar System formation, then structural mapping of the Oort cloud may provide information regarding the stellar environment in which the Sun was born. It would also provide a picture of the planetesimal population during the outer planets’ formation phase.

There are many indications that the Solar System was born in a stellar cluster. During this phase, the Sun would have been exposed to closer stellar flybys than in the Galactic field. Minor planets with a semi-major axis between ∼100 au and several 10^3^ au may still bear the signatures of the Sun being born in a $\gtrsim 1000 \mathrm{\,M_{\odot}\,pc}^{-3}$ star cluster (Brown et al. 2004; Schwamb et al. 2010). These signatures of the environment include the steep decline in material beyond 30 au, the Fe-60 and Al-26 content attributed to a closeby supernova and possibly the orbits and colours distributions of the trans-Neptunian objects (Pfalzner et al. 2024b, 2025). During the first 10–100 Myr after its formation, the Solar System’s birth cluster either dissolved or the Sun was ejected from it. Either most of the outer Oort cloud formed after the Solar System was ejected (Portegies Zwart et al. 2021), or it was severely modified during that stage (Pfalzner et al. 2024a).

The bulk of the Oort cloud originates from the region outside Jupiter’s orbit and are potentially the least thermally processed objects (Gkotsinas et al. 2024). However, the Oort cloud may also contain planetesimals that originally formed around other stars and are non-native to the Solar System. These objects would have been captured during the cluster phase, or some could be interstellar objects approaching with low relative velocity (Levison et al. 2010; Dehnen et al. 2022; Peñarrubia 2023). The relative fraction of non-native members is still an open question (Hands and Dehnen 2020; Napier et al. 2021), though none are currently known from their dynamics (Morbidelli et al. 2020).

Naturally, the cluster environment would affect potentially existing exo-Oort clouds. Oort clouds are not necessarily entirely destroyed by a close stellar flyby, but their planetesimal content is considerably reduced (Pfalzner et al. 2024a). However, the orbits of the planetesimals are strongly affected (Portegies Zwart et al. 2021), and as a consequence, an intense inward stream of exocomets is released. Thus, the cluster environment simultaneously increases the long-period exocomet rate on a short time scale, but reduces it on longer time scales, due to the reduced Oort cloud reservoir.

The existence of exo-Oort clouds around other stars is therefore theoretically possible, but lacks observational proof at present. Generally, there is the question of how comets are launched from the exo-Oort cloud region to the regions close to the host star. Wyatt et al. (2017) find that three principles maximise the cometary influx from exo-Kuiper belts: a chain of closely separated planets interior to the belt, none of which is a Jupiter-like ejector; planet masses not increasing strongly with distance and ongoing replenishment of comets, possibly by embedded low-mass planets. Thus, the structure of the planetary system strongly influences the degree of long-period exocomet influx.

## Open Questions

We close this article with a summary of questions that remain largely unanswered and thus ‘open’ for consideration. One of the most exciting aspects of astronomy as a modern science is the degree to which our growing understanding both encompasses satisfactory answers to older questions and grows a host of new questions in their place. As authors, we hope some of the readers of this chapter — perhaps even the earliest-career among us — will themselves bring answers in their turn.

- What is the role of the natal prestellar environment for the subsequently emerging star-disc system?
- How important is it for the planetesimals if the host star is a binary (with the disc in either a circumstellar or circumbinary configuration)?
- Which steps are needed to make a self-consistent model all the way from disc composition to evolved planetesimal in a reservoir?
- How well do we understand the potential differences of planetesimal formation around the Sun vs. other stars?
- Which small-body reservoir of a system is its dominant source of exocomets? Can a distinctive signature be found?
- Are planets required to produce high-eccentricity comets?
- How large is the fraction of non-local objects (captured from other stars) in an Oort cloud? Are they compositionally distinguishable?
- How massive are debris discs, which are the likely reservoirs for exocomets?
- What sets where debris discs form?
- Is the diversity seen in debris-disc shapes due to different formation conditions, or the action of planets? How do these link to exocomet production?
- How far can the analogy of debris discs being classical Kuiper belt analogues be pushed?
- What will the Vera C. Rubin Observatory’s LSST reveal about the structure of the Oort Cloud?
- Are planetesimals primarily implanted into Oort-cloud analogues during the protoplanetary disc stage or afterwards?

## Conclusions

Reservoirs of small bodies suitable for sourcing exocomets are a probable outcome of the planetesimal-formation process at any young star. While exocomets are often detected at present around A-type stars, planet formation happens at stars of a wide variety of masses. The dust grains and dust-to-gas ratio throughout a disc can vary substantially. While the planetesimal formation pathway has a range of remaining uncertainties, turbulence and oscillation within the dust-rich disc gas permit gravitational collapse and allow the growth of planetesimals. These form in huge numbers in a typical disc.

The formation history of a given system will be influenced by its stellar environment. To first order, the thermal conditions and resulting snowlines within a disc will strongly influence the composition of the planetesimals that form at that location. The mass of the star affects the distance of these snowlines, but complex additional processes can affect the available gas and ice compositions as the discs evolve with time. The presence of planets will set the dynamical architecture of the system; however, our understanding of these systems may still be affected by the current observational biases of the sample of planets around stars of different masses. The remnant solid material not incorporated into planets is visible as debris discs in the early stages, and over subsequent Myr, collisional grinding slowly depletes the populations of small bodies into dust.

Thus, at almost any main-sequence star, there will be small-body reservoirs. They present a complex mix of possibilities for exocomets to emerge. The local planetary architecture will permit a particular range of stable reservoirs, with transient populations feeding between them and swapping material out into active exocomets. The compositions of the small bodies within each reservoir will vary widely depending on their source region in the disc and the mixing history of that system.

The detailed knowledge about the small-body population of the Solar System illustrates the wealth of small-body reservoirs that can be present in a single system with relatively straightforward mass partitioning between the planets. The sheer nuance of some of these small-body populations would not be looked for in other systems if examples were absent in our system. Equally, it is always worth considering how much our detailed familiarity with this system, “the fields we know”, may lead to expectations that influence our assumptions about other systems. In general, for any planetary system, resonant relationships of the planets will sculpt and dominate the orbital stability of the small bodies, from their primordial formation locations to their eventual orbits. A given system’s current planetary architecture is only a snapshot of the range of states it may have existed in over time, and its small-body reservoirs reflect that. Several of the exocomet reservoirs described here require processes that change their orbits to turn them into an exocomet; this transformation is discussed in Mustill et al. (2025), this collection. The home of any exocomet may therefore be a transient thing.

Solar System objects provide observational constraints to the models of planetesimal formation that can be used to understand the diversity of objects around stars beyond the sun. Exploring the properties of Solar System objects with ever-more-sensitive instruments allows us to address challenging questions which test our understanding of formation processes. Even though there are many unanswered questions, the combination of the details we can observe in the Solar System with the diversity of populations around other stars will lead to an improved understanding of the processes leading to the formation of small-body reservoirs across the Galaxy.

The range of open questions for exocomet reservoirs suggests many of these details will be resolved over the next decade, as further observation by JWST and ALMA take place, and a wider range of stars are studied for exocomet observation. Furthermore, the community can look forward to new facilities as the Vera C. Rubin Observatory, NASA’s SPHEREx (Spectro-Photometer for the History of the Universe, Epoch of Reionization and Ices Explorer) and Nancy Grace Roman Space Telescope, and the Extremely Large Telescope and Giant Magellan Telescope.

## Footnotes

1 No co-orbitals are known for Mercury. Note that Trojans are not stable for Saturn or Uranus.

2 For a recent review of the composition of (162173) Ryugu sample compositions, see Yokoyama et al. (2025).
